# Level of Work Related Stress among Teachers in Elementary Schools

**DOI:** 10.3889/oamjms.2015.076

**Published:** 2015-07-01

**Authors:** Teuta Agai–Demjaha, Jovanka Karadzinska Bislimovska, Dragan Mijakoski

**Affiliations:** *Institute of Occupational Health of Republic of Macedonia, WHO Collaborating Center, Department of Occupational Medicine, Medical Faculty, Ss Cyril and Methodius University of Skopje, Skopje, Republic of Macedonia*

**Keywords:** workplace, stress, teachers, demography, job characteristics

## Abstract

**BACKGROUND:**

Teaching is considered a highly stressful occupation, with work-related stress levels among teachers being among the highest compared to other professions. Unfortunately there are very few studies regarding the levels of work-related stress among teachers in the Republic of Macedonia.

**AIM:**

To identify the level of self-perceived work-related stress among teachers in elementary schools and its relationship to gender, age, position in the workplace, the level of education and working experience.

**MATERIAL AND METHODS:**

We performed a descriptive-analytical model of a cross-sectional study that involved 300 teachers employed in nine elementary schools. Evaluation of examined subjects included completion of a specially designed questionnaire.

**RESULTS:**

We found that the majority of interviewed teachers perceive their work-related stress as moderate. The level of work-related stress was significantly high related to the gender, age, position in workplace, as well as working experience (p < 0.01), while it was significant related to level of education (p < 0.05). Significantly greater number of lower-grade teachers perceives the workplace as extremely stressful as compared to the upper-grade teachers (18.5% vs. 5.45%), while the same is true for female respondents as compared to the male ones (15.38% vs. 3.8%). In addition, our results show that teachers with university education significantly more often associate their workplace with stronger stress than their colleagues with high education (13.48% vs. 9.4%). We also found that there is no significant difference of stress levels between new and more experienced teachers.

**CONCLUSION:**

Our findings confirm that the majority of interviewed teachers perceived their work-related stress as high or very high. In terms of the relationship between the level of teachers’ stress and certain demographic and job characteristics, according to our results, the level of work-related stress has shown significantly high relation to gender, age, levels of grades taught as well as working experience, and significant relation to the level of education.

## Introduction

Each profession causes a specific level of work-related stress. Teaching as a profession is progressively becoming a rather stressful occupation [1]. Due to increased responsibilities and demanding deadlines, teaching nowadays is more stressful than ever. It has been recognized for several decades that teachers experience work-related stress on daily basis and this fact has been the focus of extensive research [2-4].

Kyriacou who has carried out varies studies on teacher stress, defines teachers’ stress as an uncomfortable feeling, negative emotion such as anger, anxiety, pressure and disappointment sourced from their work aspects as a teacher [5]. Already established high levels of stress among teachers, normally lead to work absentee and work dissatisfaction. Ultimately, teachers often opt to leave their profession [6]. Recent research has shown that one out of three teachers report teaching as being very or extremely stressful, causing the teaching profession to have the highest annual turnover rate. The annual turnover rate for teachers is 15.7% while other professions have an average annual turnover rate of 11% [7].

A number of studies have indicated the comparatively stressful nature of teaching. There are several studies that argument that teachers do experience a higher level of stress than many other professionals For instance, Pithers points out that teaching profession comparatively represents one of the most stressful jobs [8]. Kyriacou reports that teachers had the highest levels of work-related stress compared to people in other professions [9]. According to Cox et al. 78% of teachers consider work as the main source of stress in their lives while the same is true for only 38% of other professionals [10]. According to a study by Chana et al. conducted with subjects from six different professions, teachers reported the highest level of stress against lawyers, nurses, engineers, insurance agents and doctors [11]. Steadily increasing costs and consequences of teacher stress has received growing concern. To reduce the negative effects stress has on teachers, more attention needs to be placed on this growing epidemic [12].

As of today, there is very little research regarding work related stress and main stress factors among teachers in the Republic of Macedonia. The comparative paper of Eres and Atanasovska that explores the issue of work-related stress among teachers in Turkey and Macedonia represents the only study in the field so far. Their research has shown low levels of stress among teachers in Turkey and moderate levels among their colleagues in Macedonia. The study also suggests that the educational system in Macedonia has been undergoing continual changes. Consequently, the study suggests that the level of stress among teachers in Macedonia is mainly a result of the new situations and new roles that teachers face, as well as great expectations from their superiors [13].

The aim of the paper was to identify the level of self-perceived work-related stress among teachers in elementary schools and its relationship to gender, age, position in the workplace, the level of education and working experience.

## Methodology

### Study design and setting

A descriptive-analytical model of a cross-sectional study was carried out in nine elementary schools in Skopje, Republic of Macedonia. Evaluation of examined subjects included completion of a specially designed questionnaire. Out of 358 teachers to whom the questionnaire was distributed, 300 of them responded. The interviewing process was voluntary and respected the teachers’ rights to anonymity. The research was conducted between September 2013 and June 2014, in cooperation with experts from the Institute of Occupational Health of RM, WHO Collaborating Center.

### Subjects

Participants were 195 (65%) females and 105 (35%) males, aged from 26 to 64 years. According to the research data, 45% of the subjects worked as lower-grades school teachers, while 55% worked as upper-grades school teachers. Regarding the education, 159 (53%) of teachers had high education^1^, while 141 (47%) were with university (superior) degree. The shortest working experience was two years, while the longest 40 years (20.82 ± 6.94).

### Questionnaire

Prior to the research, ethical approval was granted by the Ministry of Education and Science. The questionnaire concerning the impact of stress on teachers’ health in elementary schools has been used as an instrument of the study. It was based on the UCU (University and College Union) Model Stress Questionnaire^2^ and it consists of five parts. For the purposes of the actual study we have utilized the first two parts. First part contains questions about demographic and job characteristics of the respondents (gender, age, position in the workplace, level of education and working experience). Second part consists of an item related to the level of stress at work measured with a Stress Level Scale (single item, “Please indicate your level of stress in your working place”?). To measure the level of teacher’s work-related stress, participants were asked to indicate the level of self-perceived work-related stress and/while stress levels were categorized as *low, average*, *high*, and *very high*. Finally we have analyzed the relationships between levels of teacher’s self-perceived work-related stress and different demographic and job characteristics such as gender, age, position in the workplace, level of education and working experience.

### Statistical methods

Statistical Package for the Social Science (SPSS) version 17.0 for Windows was used for data description and analysis. Categorical variables were expressed as absolute and relative numbers; quantitative variables were expressed as mean ± SD, minimal, and maximal values. The Chi-square test, t-test for independent samples, and Analysis of Variance (ANOVA) test were used to test differences in relation to the different demographic and job characteristics. P-value below 0.05 was considered statistically significant while p-value below 0.01 was considered highly significant.

## Results

The research results based on collected data are presented below. Results are shown in tables and figures and they also include additional textual description. Demographic characteristics of the subjects enrolled in the study, such as gender, age, position in the workplace, level of education and working experience, are presented in Table 1.

**Table 1:** Demographic characteristics of the study subjects

Variable	N = 300
Gender	
female	195 (65%)
male	105 (35%)
Age	
under 45	83 (27.66%)
45 +	217 (73.34%)
Position in the workplace	
teacher-lower grade	135 (45%)
teacher-upper grade	165 (55%)
Level of education	
high	159 (53%)
university (superior degree)	141 (47%)
Working experience (years) min = 2, max = 40, mean ± SD (20.82 ± 6.94)	

Note: numerical data are expressed as mean value with standard deviation; frequencies as number and percentage of study subjects with certain variable.

**Figure 1:**
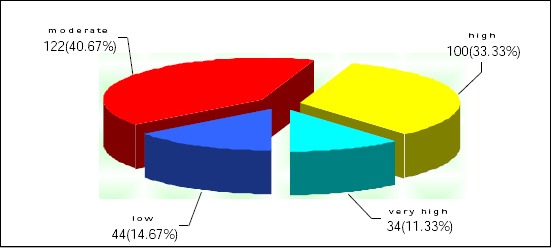
*Distribution of participants according to their level of work-related stress (Note: frequencies are expressed as number and percentage of study subjects with certain variable)*.

Figure 1 demonstrates levels of self-perceived work-related stress among teachers included in the research.

Figure 1 indicates that the majority of participants 134 (44.66%) perceived work-related stress as high 100 (33.33%) or very high 34 (11.33%), 122 (40.67%) as moderate and only 44 (14.67%) participants have reported low levels of work-related stress.

Table 2 shows that 30 (15.38%) of female respondents perceived their work-related stress as very high. On the other hand, only 4 (3.81%) of male teachers reported very high level of work-related stress. Consequently, significantly more female teachers have reported very high levels of stress as compared to male teachers.

**Table 2:** Levels of teacher’s work-related stress in relation to gender

Variable	Level of work-related stress			
	Low	Moderate	High	Very high
Gender					
female	27 (13.85%)	90 (46.15%)	48 (24.62%)	30 (15.38%)
male	17 (16.19%)	32 (30.48%)	52 (49.52%)	4 (3.81%)

Pearson Chi-square: 25.15, df=3, p = 0.000014**, p < 0.01. Note: frequencies are expressed as number and percentage of study subjects with certain variable. Tested by Chi-square test.

Table 3 shows overall levels of teacher’s self-perceived work-related stress in relation to age.

**Table 3:** Levels of teacher’s work-related stress in relation to age

Variable	Level of work-related stress			
	Low	Moderate	High	Very high
Age					
under 45	40 (48.19%)	35 (42.17%)	8 (9.64%)	0
45 +	4 (1.84%)	87 (40.09%)	92 (42.4%)	34 (15.67%)

Pearson Chi-square: 120.33, df = 3, p = 0.00000**, p < 0.01. Note: frequencies are expressed as number and percentage of study subjects with certain variable. Tested by Chi-square test.

Table 3 demonstrates that 40 (48.19%) teachers under 45 years of age perceived the work-related stress as low, while none of them rated the level of stress at the workplace as very high. On the other hand, the majority of teachers older than 45 years - 92 (42.4%) have rated the work-related stress as high, while only 4 (1.84%) as low. Clearly, our results show that significantly more teachers older than 45 years reported high or very high levels of work-related stress.

In Table 4 below, we have presented levels of teacher’s self-perceived work-related stress in relation to their position in the working place.

**Table 4:** Levels of teacher’s work-related stress in relation to position in the working place

Variable	Level of work-related stress			
	Low	Moderate	High	Very high
Position in school				
teacher lower- grade	24 (17.78%)	57 (42.22%)	29 (21.48%)	25 (18.52%)
teacher upper- grade	20 (12.12%)	65 (39.39%)	71 (43.03%)	9 (5.45%)

Pearson Chi-square: 23.29, df=3, p = 0.000035**, p < 0.01. Note: frequencies are expressed as number and percentage of study subjects with certain variable. Tested by Chi-square test.

According to our results in Table 4, significantly more lower-grade teachers perceive the workplace as very stressful as compared to the upper-grades teachers (18.5% vs. 5.45%). Consequently, there is significantly high association (p < 0.01) between work-related stress and position in the workplace.

In Table 5 below, we have presented levels of teacher’s self-perceived work-related stress in relation to the level of education.

**Table 5:** Levels of teacher’s work-related stress in relation to level of education

Variable	Level of work-related stress				
	Low	Moderate	High	Very high
Education level				
high	31 (19.5%)	56 (35.22%)	57 (35.85%)	15 (9.43%)
university	13 (9.22%)	66 (46.81%)	43 (30.5%)	19 (13.48%)

Pearson Chi-square: 9,57 df=3 p=0.023* p<0.05. Note: frequencies are expressed as number and percentage of study subjects with certain variable. Tested by Chi-square test.

Table 5 indicates that significantly more teachers with university education associate their workplace with stronger stress than their colleagues with high education (13.48% vs. 9.4%). In this case, our results show significant (p < 0.05) relationship between work-related stress and teacher’s level of education.

Finally, in Table 6 we have presented levels of teacher’s self-perceived work-related stress in relation to the working experience.

**Table 6:** Levels of teacher’s work-related stress in relation to working experience

Variable	Level of work-related stress			
	Low	Moderate	High	Very high
working experience (years)				
Mean ± SD, Rank	13.4 ± 6.4 4-40	22.24 ± 7.4 4-33	23.6 ± 4.3 2-30	17.2 ± 2.5 13-27
Rank	4-40	4-33	2-30	13-27

Analysis of Variance: F = 36.3, p = 0.000**, p < 0.01. Note: numerical data are expressed as mean value with standard deviation.

According to our results in table 6, respondents who reported lowest levels of work-related stress, they also have the lowest working experience average (13.4 ± 6.4 years). Accordingly, our results analyzed by ANOVA show significantly high (p < 0.01) association between work-related stress and teacher’s working experience.

## Discussion

Initially, our study aimed to determine the level of work related stress among teachers in selected elementary schools in Macedonia. In general, teaching has recently been considered among the most stressful professions. A study conducted by German Trade Union Confederation (DGB) in Germany during 2013, has showed that teachers, are more stressed than other professionals. According to the study, 66% of interviewed teachers suffer from certain level of work-related stress [14]. These results are in line with our findings, since 40.67% of our participants have perceived the level of stress at their workplace as moderate, additional 33.33% of them reported high levels of work-related stress, and another 11.33% have considered it as very high.

On the other hand, a research by Aftab and Khatoon from 2012 shows that 40.95% of secondary school teachers in India perceive their work-related stress as moderate, 47.70% as low stressed and only 11.35%, as highly stressful [15]. Our results are in line with these finding in terms of moderate levels of stress, since 40.67% of interviewed teachers have reported moderate levels of work-related stress. However, our results contradict findings of the research in India, since only 14.67% of the participants have reported low levels of work-related stress.

In terms of the relationship between the level of teachers’ stress and certain demographic and job characteristics, according to our results, the level of work-related stress shows significantly high relation to gender, age, levels of grades taught as well as working experience, and significant relation to the level of education.

Contrary to our study, in the research conducted by Gold and Batchelor in 2001 no differences were detected in the levels of perceived work-related stress according to gender or position in the workplace [16]. Gender differences were registered in the already mentioned study by Aftab and Khatoon from 2012, according to which, male teachers experienced higher levels of stress than female ones. Such results are contradicted by our findings according to which female respondents experience higher levels of stress than their male colleagues. They also found that, unlike our findings, teachers with high education report more work-related stress than their colleagues with university education [15].

However, a study by Anhorn conducted in 2008 suggested that working experience is a determinant in causing work-related stress among teachers. According to this study, newly employed teachers had inadequate time to complete and appropriately plan their workplace activities and workload [17]. Similarly, Smethem and Adey in 2005 have suggested that excessive workload was important obstacle for newly employed teachers to differentiate instructions and to manage classroom activities and therefore they had to bring home excessive amounts of work [18]. Again our study contradicts such findings of these authors since our results do not suggest significant difference of stress levels between new and more experienced teachers. Moreover, according to our study, respondents who believe that they are least exposed to work-related stress have on average minimum working experience. At the same time, there are no respondents aged less than 45 years of age who believe that they are exposed to significant stress in their workplace.

Main limitation of the present study is relatively small number of the interviewed subjects and the fact that only teachers from nine schools in one urban municipality were interviewed. These limitations might have certain implications on the obtained data and their interpretation. Its strength, on the other hand, lays in the fact that it is one of very few public health studies in the country that analyses levels of work-related stress among teachers and their relation with different demographic and job characteristics.

In a cross-sectional study aimed to investigate the level of self-perceived work related stress among teachers in elementary schools and its relationship to gender, age, position in the workplace, the level of education and working experience we have concluded that the majority of interviewed teachers perceive their work-related stress as high or very high. In terms of the relationship between the level of teachers’ stress and certain demographic and job characteristics, according to our results, the level of work-related stress shows significantly high relation to gender, age, levels of grades taught as well as working experience, and significant relation to the level of education.

The fact that teachers with different demographic characteristics have reported different levels of work-related stress, might serve as a guideline for future stress coping mechanisms that target specific categories of teachers. While our findings are sometime in line with other studies, in other occasions they clearly contradicting them. In general, such divergence might be due to different specific local characteristics such as mentality, standard of living and system of education. Discussions with teachers also reveal that a highly paced and rather hectic educational reform process represents one of the key challenges for teaching profession. However, to arrive to valid and accurate conclusions in this direction, it is clear that further research and analysis is needed.

## Footnotes

1 Two year college education for receiving teacher’s degree after completing the high school. Within the undergoing educational reforms in R. Macedonia this degree has to be upgraded into a university degree.

2 For more details see http://www.ucu.org.uk/2562.
